# Selective Reactivity of Anti-Japanese Encephalitis Virus NS4B Antibody Towards Different Flaviviruses

**DOI:** 10.3390/v12020212

**Published:** 2020-02-14

**Authors:** Pakieli H. Kaufusi, Alanna C. Tseng, James F. Kelley, Vivek R. Nerurkar

**Affiliations:** 1 Department of Tropical Medicine, Medical Microbiology and Pharmacology, John A. Burns School of Medicine, University of Hawaii at Manoa, Honolulu, HI 96813, USA; acytseng@hawaii.edu (A.C.T.); jkelley@hawaii.edu (J.F.K.); 2 Pacific Center for Emerging Infectious Diseases Research, John A. Burns School of Medicine, University of Hawaii at Manoa, Honolulu, HI 96813, USA; 3 Department of Molecular Biosciences and Bioengineering, College of Tropical Agriculture and Human Resources, University of Hawaii at Manoa, Honolulu, HI 96822, USA; 4 World Health Organization of the Western Pacific Region, Malaria, Other Vector-borne and Parasitic Diseases Unit, United Nations Ave, Ermita, Manila, 1000 Metro Manila, Philippines

**Keywords:** flavivirus, West Nile virus, Japanese encephalitis virus, NS4B, JEV NS4B antibody, cross-reactivity, immunoassays

## Abstract

Studies investigating West Nile virus (WNV) NS4B protein function are hindered by the lack of an antibody recognizing WNV NS4B protein. Few laboratories have produced WNV NS4B antibodies, and none have been shown to work consistently. In this report, we describe a NS4B antibody against Japanese encephalitis virus (JEV) NS4B protein that cross-reacts with the NS4B protein of WNV but not of dengue virus (DENV). This JEV NS4B antibody not only recognizes WNV NS4B in infected cells, but also recognizes the NS4B protein expressed using transfection. It is evident from this data that the JEV NS4B antibody is specific to NS4B of WNV but not to NS4B of the four DENV serotypes. The specificity of this antibody may be due to the notable differences that exist between the amino acid sequence identity and antigenic relationships within the NS4B protein of the WNV, DENV, and JEV.

## 1. Introduction

The West Nile virus (WNV) genome consists of a single-stranded, positive-sense RNA of approximately 11-kb that encodes a single polyprotein precursor, which is processed by cellular and viral-encoded proteases into three structural proteins [capsid (C), pre-membrane (prM), envelope (E)] and seven nonstructural (NS) proteins [NS1, NS2A, NS2B, NS3, NS4A, NS4B, NS5]. WNV, Japanese encephalitis virus (JEV) and the four serotypes of dengue virus (DENV) all belong to the genus *Flavivirus*, but are grouped further into different serocomplexes based on their antigenic relationships revealed by neutralization assays [1]. The four DENV serotypes belong in the DENV serogroup while WNV is one of the ten recognized members (JEV, WNV, Kunjin, Alfuy, Koutango, Kokobera, Murray Valley encephalitis, Stratford, Usutu, St. Louis encephalitis) of the JEV serogroup [2]. The serogroup is based on the recognition of the E proteins of these viruses. 

Cross-reactivity of species within or between serogroups in serological tests is a common feature of flaviviruses. These cross-reactive responses make the interpretation of serological tests such as, enzyme-linked immunosorbent assay (ELISA) and neutralization tests, difficult [3]. Antibodies against the E protein of JEV could cross-react with members of JEV serogroup as well as with some viruses outside the JEV serogroup, such as DENV [4]. NS1 was found to be more specific than the E protein for serological testing and some antibody-based epitope-blocking ELISAs have been developed to differentiate between JEV, DENV and WNV infections in humans [5]. Antibodies to a linear epitope on the prM of JEV shows no cross-reactivity with either WNV or DENV, suggesting that prM can be applied as a potential antigen for species-specific assays [6]. 

WNV NS4B is the largest of the hydrophobic NS proteins of flaviviruses and consists of three endoplasmic reticulum (ER) membrane-spanning segments [7] but its exact role in the virus life cycle is not known. The NS4B of DENV also spans the ER and is part of the membrane-bound viral replication complexes [8]. NS4B is conserved in the Flaviviridae family and can induce alterations of the ER membrane to form distinct membrane structures, which provide a platform for the viral replication complexes [9]. Despite its ability to inhibit the host interferon (IFN) antiviral response [10,11] and induce monocyte-derived inflammatory cytokines [12], NS4B may play a more direct role in viral RNA replication and pathogenesis as suggested by numerous NS4B mutational studies [13,14,15,16,17]. Therefore, further exploring the role of NS4B will undoubtedly reveal new insights into WNV pathogenesis. However, the absence of a readily available WNV NS4B antibody largely restricts the study of this protein. Efforts from some laboratories to produce dependable WNV NS4B antibodies have been unsuccessful, and a versatile antibody is needed for the detection of WNV NS4B in a variety of assays [18]. In this report, we describe a NS4B antibody against JEV NS4B protein that cross-reacts with NS4B protein of WNV but not of DENV.

## 2. Materials and Methods 

### 2.1. Cells and Virus

Low passage (6–11) HEK293 (ATCC CRL-1573) and Vero cells (ATCC CCL-81) were cultivated in Dulbecco’s modified Eagle medium (DMEM) (Cat#D5546, Millipore Sigma, Burlington, MA, USA) supplemented with 10% fetal bovine serum (FBS) (Cat#MT35015CV, ThermoFisher Scientific, Waltham, MA, USA) and M199 media (Cat#11150059, ThermoFisher Scientific, Waltham, MA, USA) supplemented with 5% FBS, respectively. WNV_NY99_ (NY99 strain) used in this study was originally isolated from crow brain and passaged once in Vero cells. HEK293 or Vero cells were infected with WNV_NY99_ at a multiplicity of infection (MOI) of 1. Vero cells were infected with DENV serotypes (DENV-1 (Hawaii strain), DENV-2 (NGC strain), DENV-3 (CH53489), DENV-4 (H241 strain), or the Nakayama strain of JEV at a MOI of 2. 

### 2.2. Plasmid Constructs

Standard molecular biology techniques were used for cloning [19]. Briefly, viral RNA was extracted from the supernatant of Vero cells infected with WNV NY99 strain using the QIAamp Viral RNA mini kit (Cat#52904, Qiagen, Hilden, Germany). Extracted RNA was used as a template to generate cDNA with the SuperScript IV first-strand synthesis kit (Cat#18091050, ThermoFisher Scientific, Waltham, MA, USA). The cDNA was used as template for PCR with the NS4B primer pairs as described previously [9]. The PCR-amplified WNV NS4B gene was either fused to the Cycle 3 green fluorescent protein (GFP) in TA cloning vectors pcDNA3.1/CT/NT-GFP-TOPO (NS4B-GFP plasmid) or to a V5 epitope in a pcDNA3.1/V5-His expression vector (NS4B-V5/His plasmid) from Invitrogen as described previously [9]. The resulting WNV NS4B-GFP and NS4B-V5/His plasmids (Supplementary Figure S2) were verified by restriction enzyme digest and sequencing. Analysis of the plasmid DNA sequence was performed using DNASTAR Lasergene 7.1 Sequence Analysis software (Madison, WI, USA).

### 2.3. Transient Transfections and Protein Expression

PolyFect Transfection reagent (Cat#301105, Qiagen, Hilden, Germany), a lipid-based reagent, was used to conduct transient transfections in 24-well plates, 6-well plates or coverslips with 1.0 μg of plasmid DNA per 2.5 × 10^5^ cells, according to the manufacturer’s protocol. Briefly, the plasmid DNA was mixed with the Polyfect reagent in DMEM without FBS for 10 min, diluted with an appropriate amount of growth medium, and the resulting DNA-Polyfect complexes were added to the seeded HEK293 cells grown to 70–80% confluency. Twenty-four hours after transfection, the cells were either fixed with 3.7% paraformaldehyde (PFA) for immunofluorescence labeling or harvested for western blot (WB) assays.

### 2.4. Antibodies

The anti-Japanese encephalitis virus (JEV) NS4B antibody (Cat#GTX125865) was procured from GeneTex, Inc. (Irvine, CA, USA) at a concentration of 0.76 mg/mL in storage buffer (0.1M Tris, 0.1M Glycine, 20% Glycerol (pH 7) with 0.01% thimerosal as a preservative). Briefly, the antibody was generated by GeneTex, Inc as follows: the full length recombinant JEV NS4B protein (255 amino acids in length) was derived from the JEV Jaoars982 strain (GenBank accession # NP_775673.1). The JEV NS4B protein was purified by Ni Sepharose 6 Fast Flow histidine-tagged protein purification resin. This purified protein was used as an immunogen for antibody production. Serum collected from immunized rabbits was subjected to antigen-affinity chromatography using the purified NS4B recombinant protein as the antigen to isolate anti-JEV NS4B rabbit polyclonal IgG isotype antibody. This unconjugated rabbit IgG polyclonal antibody was validated for immunocytochemistry (ICC), immunofluorescence (IFA), and WB assays, but was not tested for use in other applications. The suggested optimal dilutions were 1:100–1:2000 for ICC/IFA, and 1:500–1:3000 for WB. This antibody has been utilized to study JEV [20], but not other members of the genus *Flavivirus* such as West Nile virus (WNV), dengue (DENV), and Zika virus (ZIKV). For the detection of NS4B protein in the present study, the anti-JEV NS4B antibody was diluted 1:150 (5.1 µg/mL) or 1:1500 (0.5 µg/mL) for IFA and WB assays, respectively. 

The anti-WNV NS1 and anti-WNV Env mouse monoclonal antibodies were kindly provided by Dr. Michael S. Diamond (Washington University in St. Louis, Saint Louis, MO, USA). The anti-flavivirus dsRNA mouse monoclonal antibody (J2 monoclonal antibody, Cat#10010200) was purchased from the English & Scientific Consulting in Hungary. Primary and secondary antibodies used for immunostaining and WB assays were diluted as described previously [9].

### 2.5. Indirect Immunofluorescence Test

For the detection of WNV NS4B in infected or transfected cells, HEK293 or Vero cells were fixed with 3.7% PFA in 1X PBS and permeabilized in 0.4% Triton X-100. The fixed cells were then incubated with the anti-JEV NS4B antibody at 1:150 dilution followed by a goat anti-rabbit IgG Alexa Fluor 488 secondary antibody at 1:500 dilution or the goat anti-rabbit IgG Alexa Fluor 555 secondary antibody at 1:400 dilution (Supplementary Figures S1 and S2). For detection of other WNV proteins, the fixed cells were incubated with anti-WNV Env (1:100 dilution), anti-WNV NS1 (1:100 dilution) or anti-flavivirus dsRNA (1:100 dilution) followed by the goat anti-mouse IgG Alexa Fluor 555 (1:400 dilution) or the goat anti-mouse IgG Alexa Fluor 488 (1:500 dilution) (Figure S1). For co-detection of the WNV NS4B protein in the transfected cells, the fixed cells were incubated with mouse anti-V5/His monoclonal antibody (1:100 dilution) or rabbit anti-GFP polyclonal antibody (1:100 dilution) followed by goat anti-mouse IgG Alexa Fluor 488 at 1:500 dilution (Supplementary Figure S1) or goat anti-rabbit IgG Alexa Fluor 555 at 1:400 dilution, respectively (Supplementary Figure S2). Slides were viewed and fluorescence images were captured using Olympus confocal microscope. For quantitation of co-localization between JEV NS4B and other viral proteins in infected cells, the number of JEV NS4B-positive cells was counted and converted into a percentage of the total number of WNV Env-positive, NS1-positive or dsRNA-positive cells per field. Ten to 15 microscopic fields, each containing 15 to 30 infected cells per treatment were counted. The efficiency of infection per treatment was also calculated by dividing the number of infected cells (as indicated by WNV Env, WNV NS1 or dsRNA staining) by the total number of DAPI-stained cells in the field. The slides were viewed, and images were captured with 40× objective and the co-localized cells were confirmed with a 63× objective. The images were processed (image, adjustments, and levels) with the Adobe Photoshop CS3 Version 10.0.1 according to the policy formulated by the Digital Image Processing & Ethics Group of the Microscopy Society of America (MSA) Education Committee.

### 2.6. Cell Lysis

The infected or transfected cells were trypsinized and washed with ice-cold 1X PBS in pre-cooled microcentrifuge tubes and lysed with ice-cold lysis buffer (Cat#78503, ThermoFisher Scientific, Waltham, MA, USA) containing 1% protease inhibitor (0.5 mL per 5 × 10^6^ cells in 60 mm dish or 75 cm^2^ flask) for 2 h at room temperature or 4 °C overnight with gentle shaking. The microcentrifuge tube containing the lysate was centrifuged at 14,000 rpm at 4 °C for 45 min to pellet the cellular debris. The supernatant was transferred into a clean chilled microcentrifuge tube, kept on ice, and the pellet was discarded. The protein concentration was determined using a Quick Start^TM^ Bradford Protein Assay kit (Cat#5000201, Bio-Rad, Hercules, CA, USA), and bovine serum albumin (BSA) was used as the protein standard. The lysates were then frozen at −80 °C for protein analysis using SDS-PAGE.

### 2.7. Western Blotting Assay

Approximately 40–50 g total protein was mixed with 1× NuPage LDS sample buffer (Cat#NP0007) and separated using 4–12% precast NuPAGE gels (Cat#NW00BOX, ThermoFisher Scientific, Waltham, MA, USA). Following SDS-PAGE, the proteins were transferred onto a nitrocellulose membrane according to the manufacturer’s instructions. The membrane was blocked with 2% bovine serum albumin in 1× PBST and incubated with the appropriate primary antibody diluted in 1× PBST buffer followed by incubation with either alkaline phosphatase (AP)-conjugated or horseradish peroxidase (HRP)-conjugated secondary antibody. After washing 3–6 times with PBST buffer, the signals were detected using a chemiluminescence system.

### 2.8. Hydrophobicity and Protein Sequence Alignment Analyses

The primary amino acid sequence of NS4B of the following selected members of the flaviviruses were obtained from NCBI: DENV-1 (EU848545.1); DENV-2 (EF457904.1); DENV-3 (M93130.1); DENV-4 (AY947539.1); WNV (YP_001527886); JEV (NP_775673). The secondary structure and membrane spanning residues of NS4B for different flaviviruses were predicted using SOSUI (http://harrier.nagahama-i-bio.ac.jp/sosui/sosui_submit.html). The amino acid sequence of NS4B of WNV and DENV 1–4 were compared to JEV NS4B and alignment summary scores were calculated using the ClustalW multiple sequence alignment program (http://www.ebi.ac.uk/Tools/msa/clustalw2).

## 3. Results

### 3.1. JEV NS4B Antibody Detects NS4B Protein of WNV_NY99_ but Not of the Four DENV Serotypes

**Figure 1:**
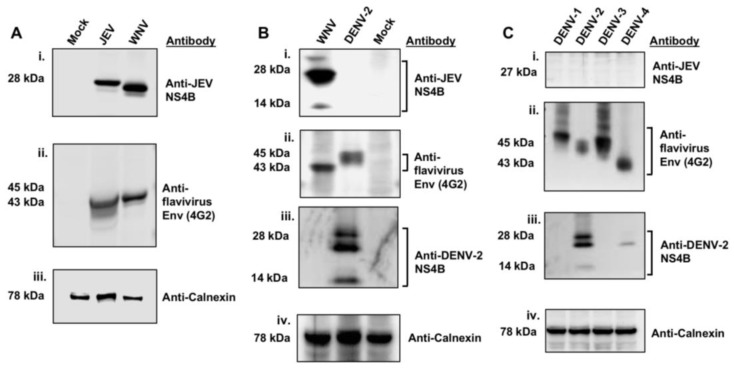
Detection of WNV_NY99_ NS4B with the anti-JEV NS4B antibody. Vero cells were mock-infected or infected with (**A**) JEV (MOI of 2) and WNV_NY99_ (MOI of 1), (**B**) WNV_NY99_ (MOI of 1) and DENV-2 (MOI of 2) or (**C**) DENV serotypes 1, 2, 3, 4 (MOI of 2) and harvested 24 h after infection. Vero cell lysates (50 μg) were separated by 4–12% SDS-PAGE and western blot analyses was performed with antibodies against JEV NS4B, flavivirus Env, DENV-2 NS4B and calnexin, as indicated on the right side of each panel. Calnexin served as an internal loading control and the presence of the Env protein was used to confirm flavivirus infection. Molecular weights (kDa) are given on the left side of each panel. Abbreviations: DENV-1: Dengue 1; DENV-2: Dengue 2; DENV-3: Dengue 3; DENV-4: Dengue 4; Env: Envelope.

The cross-reactivity of the anti-JEV NS4B antibody procured from GeneTex, Inc. was tested by WB analysis of total protein lysates from Vero cells infected with the Nakayama strain of JEV, the NY99 strain of WNV, and the NGC strain of DENV-2. Infected Vero cells revealed bands of approximately 28- and 27-kDa in sizes in JEV and WNV lysates (Figure 1A-i), respectively, but not in DENV-2 lysate (Figure 1B-i) when probed with JEV NS4B antibody. The 27-kDa band in WNV lysate is consistent with the published data [9,21]. In addition, the 27-kDa protein detected in WNV infected HEK 293 cell lysates, however, was completely undetectable in uninfected control cell lysates at 24 hr after-infection using the anti-JEV NS4B antibody (Supplementary Figure S1B), indicating that the 27-kDa band is the NS4B protein. Since NS4B protein was not detected in the DENV-2 lysate using the anti-JEV NS4B antibody, it is possible that infection of Vero cells was either inefficient or cells were not infected at all. To test this possibility, we incubated the same immobilized membrane with flavivirus 4G2 antibody, a monoclonal antibody that recognizes the E protein of most members of the genus *Flavivirus*. Expected bands of approximately 43- and 45-kDa in size [22] were detected in WNV and DENV-2 lysates, respectively, (Figure 1B-ii) indicating that the Vero cells were indeed infected with DENV-2. Additionally, the DENV-2 infection of Vero cells was confirmed using an antibody to DENV-2 NS4B protein (Figure 1B-iii). An approximately 78-kDa band corresponding to the host ER-localized protein, calnexin was detected in all lysates (Figure 1A-iii,B-iv) with about the same intensity, demonstrating that the equivalent amount of protein was loaded.

We further analyzed the total lysates of Vero cells infected with each of the four DENV serotypes [DENV1 (Hawaii strain), DENV2 (NGC strain), DENV3 (CH53489), DENV4 (H241 strain)] using the anti-JEV NS4B antibody. The NS4B band was not detected in all four DENV serotypes, (Figure 1C-i) however, the host protein calnexin was detected in the DENV serotypes (Figure 1C-iv). The protein membrane was also incubated with a DENV-2 NS4B antibody and revealed similar bands consistent with that observed in Figure 1B-iii (Figure 1C-iii). Interestingly, DENV-2 NS4B antibody cross-reacted weakly with DENV-4 NS4B (Figure 1C-iii). The infection of Vero cells by the DENV serotypes was confirmed with the detection of DENV envelope protein in the cell lysates using the 4G2 antibody (Figure 1C-ii). These data demonstrated that the Vero cells were infected with DENV serotypes and that the anti-JEV NS4B antibody selectively detected NS4B protein in the WNV infected lysate but not in the lysates of each of the four DENV serotypes.

### 3.2. Assessment of the Distribution and Localization of Intracellular WNV_NY99_ NS4B Protein to Other Viral Replication Components

**Figure 2:**
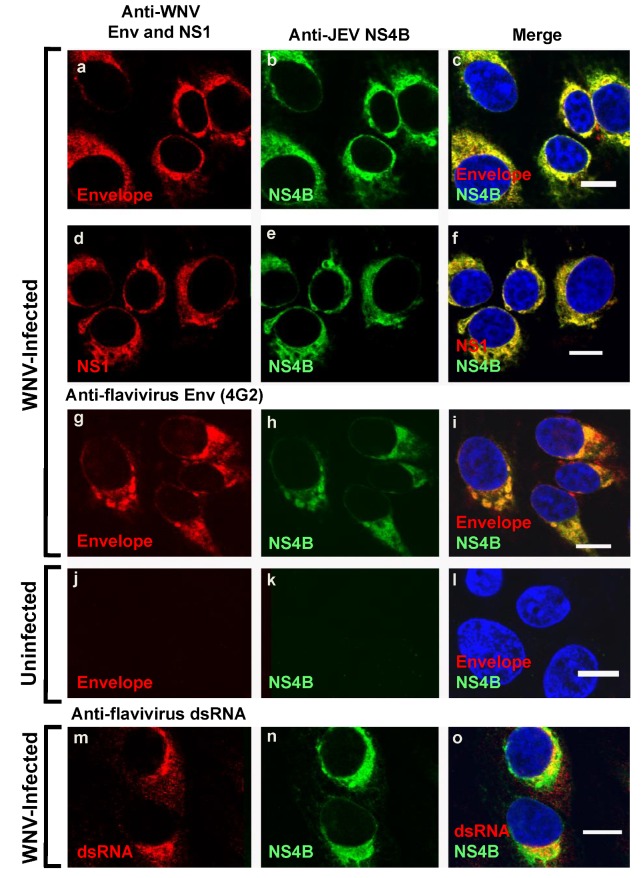
Colocalization of WNV_NY99_ NS4B with Env protein, NS1 protein, and viral dsRNA in WNV-infected cells. Vero cells were infected with WNV_NY99_ at a MOI of 1 (**a**–**i**,**m**–**o**) or mock infected (**j**–**l**). At 24 h after infection, cells were fixed with 3.7% paraformaldehyde and processed for immunofluorescence (IF). Each panel represents the co-localization of cells (100% co-localization) in 10 to 15 fields in two independent infection experiments, with an average infection efficiency of 15%. Antibodies used for immunostaining are depicted on the top of the panels. Nuclear DNA was stained with 4,6-diamidino-2-phenylindole (DAPI). Merged pictures are shown on the right (**c**,**f**,**i**,**l**,**o**). Slides were analyzed by confocal laser scanning microscopy. Confocal microscopy images were of optical slice thickness ~1 μm. Scale bar, 10 μm. Abbreviations: WNV: West Nile virus NY99 strain (WNV_NY99_); JEV: Japanese encephalitis virus; NS1: Non-structural protein 1; NS4B: Non-structural protein 4B.

We further explored whether the WNV NS4B protein identified by the anti-JEV NS4B antibody (Figure 1A-i,1B-i) was also associated with other known viral components of the flavivirus replication complexes [8,23,24]. We and others have previously demonstrated that the elements of the flavivirus replication complexes include the replicating double-stranded RNA (dsRNA) [8,25], NS1 protein [26,27], envelope protein [28] and the NS4B protein [9]. Consistent with these results, our data using triple-channel confocal imaging of WNV infected Vero cells stained with anti-JEV NS4B, WNV Env, NS1, flavivirus 4G2 and dsRNA antibodies demonstrated the co-localization (Figure 2) of NS4B with the envelope protein (a–c and g–i), NS1 protein (d–f), and dsRNA (m–o). The same immunostaining was repeated in WNV infected HEK293 cells and similar results were observed (Figure S1A). In uninfected cells, no red or green fluorescence was observed except for the nuclear staining (Figure 2j–l). The confocal microscopy images confirm that the anti-JEV NS4B antibody detected WNV_NY99_ NS4B protein in the viral replication complexes in both WNV infected Vero and HEK293 cells and the detection is not cell-type specific.

### 3.3. Detection of NS4B Protein Harbored in the NS4B C-Terminal Tagged Plasmids with JEV NS4B Antibody

**Figure 3:**
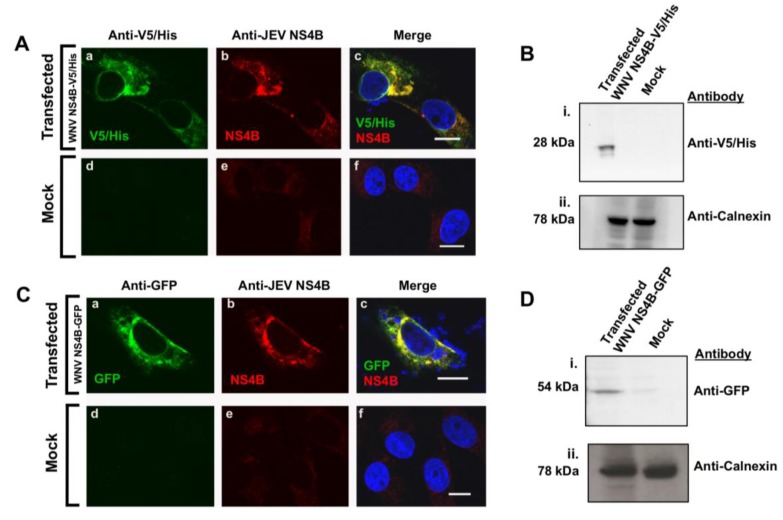
Detection of WNV_NY99_ NS4B with the anti-JEV NS4B antibody in transfected cells. Vero cells were transfected with (**A**,**B**) WNV_NY99_ NS4B-V5/His fusion, (**C**,**D**) NS4B–GFP fusion plasmids or mock transfected ((**A**) d–f; (**C**) d–f). At 24 h after transfection, cells were fixed and processed for IF (**A**,**C**) or lysed for western blot analyses (**B**,**D**). (**A**,**C**) Antibodies used for immuonolabeling are listed on the top of each panel and the detected fusion (V5/His or GFP) or viral (NS4B) proteins are depicted in the lower left corner of each image. Nuclear DNA was stained with DAPI. Merged images are shown on the right (c and f). Slides were analyzed by confocal laser scanning microscopy. Confocal microscopy images were of optical slice thickness ~1 μm. Scale bar, 10 μm. (**B**,**D**) Total cell lysates (50 μg) from NS4B-V5/His or NS4B-GFP transfected Vero cells were separated by 4–12% SDS-PAGE gel followed by immunoblotting with anti-V5/His or anti-GFP antibodies as shown on the right side of each panel. Calnexin served as an internal loading control. Molecular weights (kDa) are given on the left side of each panel. Abbreviations: V5/His: V5/Histidine tags; GFP: Green fluorescent protein tag.

A V5/His tag (Figure 3A,B) or GFP protein (Figure 3C,D) located at the C-terminal end of WNV_NY99_ NS4B protein were constructed to facilitate detection during expression. The plasmids were transfected into Vero cells (Figure 3) or HEK293 cells (Supplementary Figure S2) for immunostaining analyses. The expressed NS4B protein was expected to be detected by the anti-V5 or anti-GFP antibodies or with the anti-JEV NS4B antibody. We conducted a co-localization assay on fixed cells by confocal IF microscopy at 24 h after transfection (Figure 3A,C; Supplementary Figure S2A). Triple-channel confocal imaging demonstrated perfect overlap between the red (anti-JEV NS4B) and the green fluorophores (anti-V5/His) in fixed Vero cells transfected with the NS4B-V5/His plasmid indicating that the same protein was stained, with the blue-fluorescent DAPI in the nucleus (Figure 3A(a–c)). In the control cells, neither the red nor the green fluorescent labels were detected except for the blue-fluorescent DAPI in the nucleus (Figure 3A(d–f)). A comparable overlapping between the red and the green fluorophores was also observed in fixed cells transfected with the NS4B-GFP plasmid (Figure 3C(a–c)) and none of these fluorophores were detected in the untransfected cells other than the blue-fluorescent DAPI in the nucleus (Figure 3C(d–f)). Both V5/His tag and GFP protein tagged to NS4B protein expressed in HEK293 cells was detected by the anti-JEV NS4B antibody (Supplementary Figure S2A), ruling out the possibility of cell-type specificity.

WB analysis was also conducted on the total protein lysates of the transfected cells (Figure 3B,D). The nitrocellulose membrane incubated with anti-V5/His antibody (Figure 3B(i)) or anti-GFP antibody (Figure 3D(i)) revealed the expected sizes of approximately 28 kDa or 52 kDa bands for NS4B-V5/His and NS4B-GFP, respectively. No band was detected in the control cell lysate (Figure 3B(i),D(i)). In these membranes, calnexin was detected in both cell lysates demonstrating that equivalent amount of protein was loaded on the gel (Figure 3B(ii),D(ii)). WB analysis of WNV NS4B-V5/His transfected HEK293 cells also detected NS4B protein by the anti-JEV NS4B antibody, further confirming that this detection was not cell-type specific (Supplementary Figure S2).

Taken together, the confocal IF microscopy and WB data demonstrated that the anti-JEV NS4B antibody can detect NS4B protein regardless of the fused tags and further confirmed that the identified protein in lysates of the WNV infected cells was indeed the NS4B protein. 

### 3.4. In Silico Analyses of Genus Flavivirus NS4B Protein

**Figure 4:**
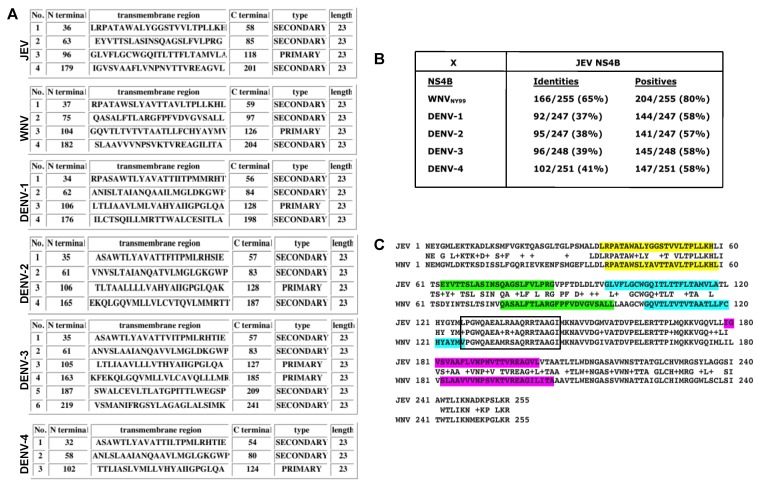
Hydrophobicity and multiple sequence alignment analyses of NS4B of different flaviviruses. (**A**) Summary of transmembrane prediction results generated by the SOSUI program showing the number, positions, residues, length, and type of each of the transmembrane helices. (**B**) Alignment summary scores after multiple sequence alignment of NS4B proteins of WNV and four serotypes of dengue against JEV using ClustalW2 program. (**C**) Complete NS4B amino acid alignments of the WNV NY99 and JEV Nakayama strains. Highlighted residues in different colors depict the predicted location of the four transmembrane helices. The boxed region depicts the highly conserved region among flaviviruses. Virus abbreviations and (Genbank accession number): DENV-1: Dengue 1 (EU848545.1); DENV-2: Dengue 2 (EF457904.1); DENV-3: Dengue 3 (M93130.1); DENV-4: Dengue 4 (AY947539.1); WNV: West Nile virus NY99 strain (YP_001527886); JEV: Japanese encephalitis (NP_775673).

To understand why the anti-JEV NS4B antibody detected WNV NS4B protein but not the NS4B of the four DENV serotypes, we conducted hydrophobicity analysis and multiple alignments of the NS4B of JEV, WNV, and the four DENV serotypes. Hydrophobicity analysis utilizing predictions generated by the SOSUI program [29] demonstrated the numbers, positions, types, and lengths of transmembrane helices for each NS4B protein from JEV, WNV, and the four serotypes of DENV (Figure 4A). It is evident from this analysis that the numbers, positions, and types are quite variable except for the length of the transmembrane helices among JEV, WNV and four DENV serotypes (Figure 4A). Although JEV, WNV, DENV-1, and DENV-2 NS4B have four transmembrane helices compared to DENV-3 NS4B with six and DENV-4 with three, it is interesting to note that the positions of each of the first four transmembrane helices are located at about the same region within the NS4B proteins (Figure 4A). The amino acid identity of WNV to JEV NS4B protein is much higher, about 65%, compared to the four DENV serotypes, ranging from 37% to 41%, based on the ClustalW2 multiple alignment software (Figure 4B, Supplementary Figure S3). Also, out of the four transmembrane helices only the first transmembrane helix is highly conserved between WNV and JEV (Figure 4C, color-coded regions). The regions that appear to completely protrude out of the membrane are located at the N- and C-terminal ends of NS4B as well as the central 52-aa region following the third transmembrane helix (Figure 4C, non-highlighted regions). Interestingly, this central region is highly conserved between WNV and JEV (approximately 88% identity), indicating that it may be the potential epitope recognized by the anti-JEV NS4B antibody.

## 4. Discussion

It is a characteristic feature of antibodies against the E and NS1 proteins of JEV to cross-react with other viruses in the JE serogroup as well as with some viruses outside the JE serogroup, such as the DENV [1,4]. The anti-flavivirus group-antigen antibody (4G2) cross reactivity is confirmed in this study since 4G2 antibody detected the E proteins of JEV, WNV and four serotypes of DENV. 

Using an antibody against NS4B protein of JEV, we found that it cross-reacted with the NS4B protein of WNV but not of DENV in all analytical assays we employed. Using high-resolution confocal microscopy on fixed WNV infected cells, the anti-JEV NS4B antibody detected the WNV NS4B protein. Components of the WNV replication complexes, including the replicating double-stranded RNA (dsRNA), the NS1, and E proteins, were localized in the infected cells as demonstrated previously by our group [9]. 

We then investigated NS4B protein in WNV or DENV-infected cell lysates with WB. The NS4B protein of approximately 27-kDa, also the predicted size, was recognized by anti-JEV NS4B antibody in WNV lysate but not in DENV lysates. Similar immunostaining assays were conducted on the cells expressing the WNV NS4B protein harbored in an expression plasmid. The results revealed that the detected protein in the WNV infected cells, as well as the protein expressed in our plasmid system, is indeed the NS4B protein. 

In silico analysis of the JEV NS4B protein sequence with the NS4B sequences of WNV and the four serotypes of DENV, as expected, indicated that the JEV and WNV are closely related. The NS4B regions of JEV and WNV that are highly homologous are located in the first transmembrane helix and in the membrane-protruding region following the third transmembrane helix. This in silico data suggest that the epitope recognized by the JEV NS4B antibody may be located in these highly conserved regions of the NS4B protein between WNV and JEV. Future studies are warranted to further decipher the conserved regions of WNV and JEV that are recognized by the anti-JEV NS4B antibody. 

## 5. Conclusions

In summary, the cross-reactive JEV NS4B antibody will serve as a useful reagent for laboratory research to gain valuable knowledge into a more direct role that NS4B may play in WNV RNA replication and pathogenesis. This JEV NS4B antibody can also potentially be utilized in screening assays to differentiate between WNV and DENV, which co-circulate in Southeast Asia.

## Supplementary Materials

Supplementary materials can be found at https://www.mdpi.com/1999-4915/12/2/212/s1.
