# Supplementary material for: Selective Reactivity of Anti-Japanese Encephalitis Virus NS4B Antibody Towards Different Flaviviruses
Source: Viruses. 2020 Feb 14;12(2):212. doi: 10.3390/v12020212 (PMC7077296; doi:10.3390/v12020212)
Supplement: Supplementary file 1 [file viruses-12-00212-s001.pdf]

Supplementary Materials

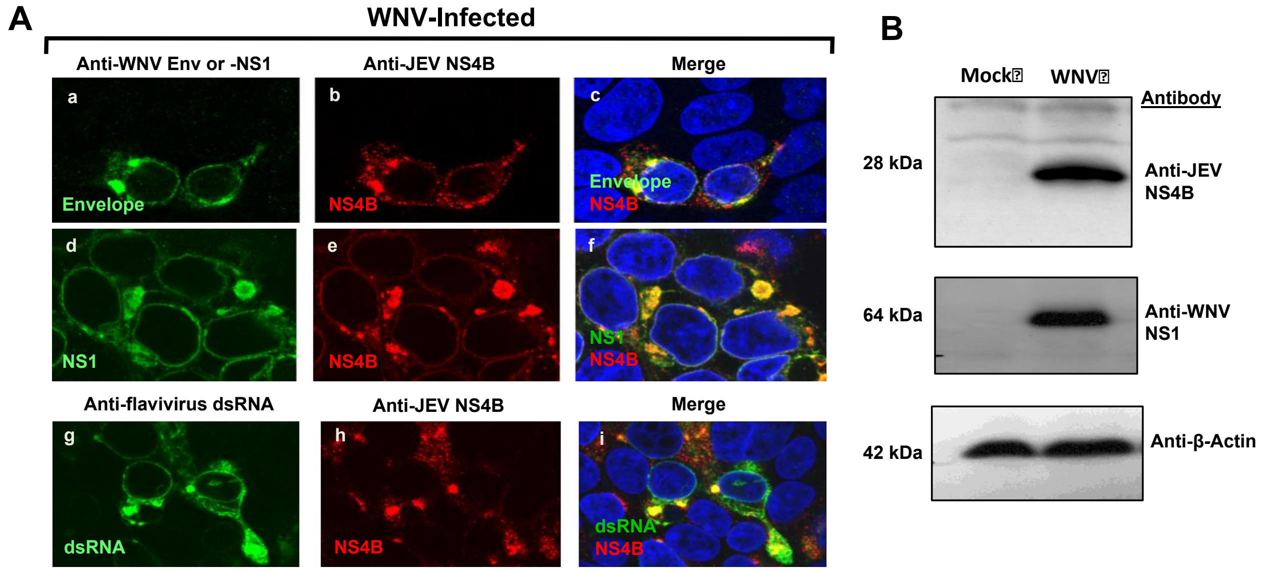

Figure S1. Detection of WNV_{NY99} NS4B in WNV infected HEK293 cells with the anti-JEV NS4B antibody. HEK293 cells were infected with WNV_{NY99} at a MOI of 1. Twenty-four hours after infection, cells were fixed for immunofluorescence or lysed for Western blot analysis. (A) Confocal microscopy images showing the colocalization of WNV_{NY99} NS4B with WNV Env and NS1 proteins (a and d) and dsRNA (g) in infected HEK293 cells. The antibodies used for immunolabeling are depicted on the top of each panel and the detected viral components (Env, NS1 or dsRNA) are depicted in the lower left of each image. DAPI was used to stain nuclear DNA. Merged pictures are shown on the right (c, f, and i). Confocal images were of optical slice thickness ~1 µm. (B) Western blot detection of WNV_{NY99} NS4B by the anti-JEV NS4B antibody in infected HEK293 cells. The antibodies used for immunoblotting are shown on the right side of each panel. An anti-WNV NS1 antibody was used to confirm infection. ß-actin served as the loading control. Molecular weight markers (kDa) are given on the left side of each panel. Abbreviations: WNV: West Nile virus NY99 strain (WNV_{NY99}); JEV: Japanese encephalitis; NS4B: Non-structural 4B protein.

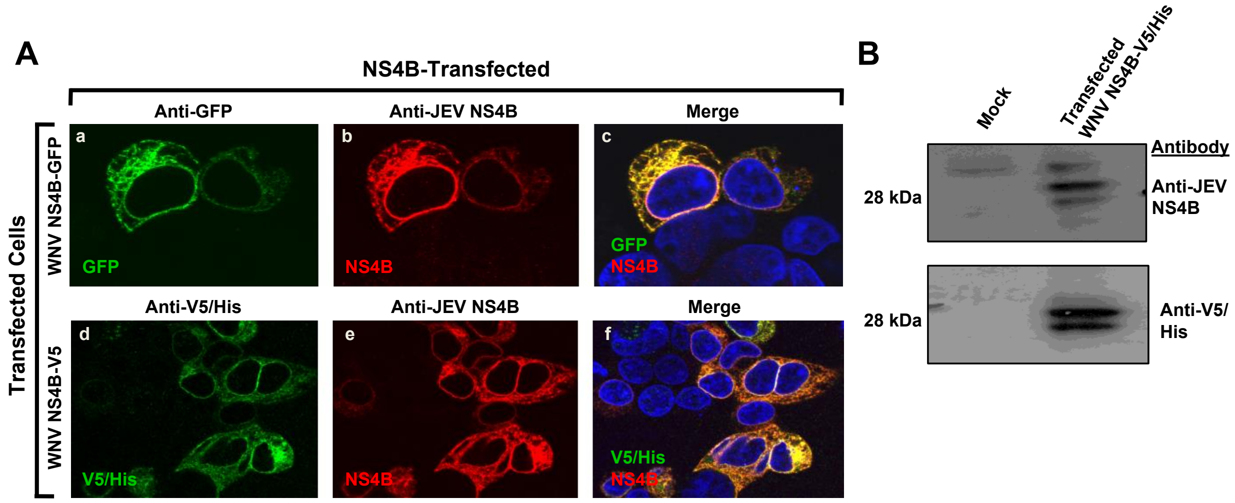

Figure S2. Detection of WNV_{NY99} NS4B with the anti-JEV NS4B antibody in transfected HEK293 cells. (A) Cells were transfected with WNV_{NY99} NS4B-GFP fusion (a, b and c) or NS4B-V5/His fusion (d, e and f) plasmids. Twenty-four hours after transfection, cells were fixed and processed for immunofluorescence. Antibodies used in this study are depicted on the top of each panel and the detected fusion (GFP or V5/His) or viral (NS4B) proteins are indicated in the lower left of each image. Nuclear DNA was counterstained with DAPI. Merged images are shown on the right (c and f). Slides were analyzed by confocal laser scanning microscopy. Confocal IF images were of optical slice thickness ~1 µm. B) HEK293 cells transfected with WNV_{NY99} NS4B-V5/His fusion plasmid were harvested 24 hours after transfection. Cell lysates (50 µg) were subjected to SDS-PAGE and Western blot analysis using anti-JEV NS4B and anti-V5/His antibodies. Molecular weight (kDa) is given on the left side of each panel. Abbreviations: V5/His: V5/Histidine tags; GFP: Green fluorescent protein tag.

```
DENV-1          NEMGLLETTKKDLG---IGHVAVENHHHATMLDVDLRPASAWTLYAVATTIITPMMRHTI 57
DENV-3          NEMGLLETTKRDLG---MSKEPGVVSP-TSYLDVDLHPASAWTLYAVATTVITPMLRHTI 56
DENV-2          NEMGFLEKTKKDLG---LGG-STMQQPEINILDIDLRPASAWTLYAVATTFITPMLRHSI 56
DENV-4          NEMGLIEKTKTDFG---FYQ----VKTETTILDVDLRPASAWTLYAVATTILTPMLRHTI 53
JEV             NEYGMLEKTKADLKSMFVGKTQASGLTGLPSMALDLRPATAWALYGGSTVVLTPLLKHLI 60
WNV             NEMGWLDKTKSDISSLFGQRIEVKENFSMGEFLLDLRPATAWSLYAVTTAVLTPLLKHLI 60
                ** * ::.** *:                  : :**:**:**:**. :*..:**:::* *

DENV-1          ENTTANISLTAIANQAAILMGLDKGWPISKMDIGVPLLALGCYSQVNPLTLIAAVLMLVA 117
DENV-3          ENSTANVSLAAIANQAVVLMGLDKGWPISKMDLGVPLLALGCYSQVNPLTLIAAVLLLVT 116
DENV-2          ENSSVNVSLTAIANQATVLMGLGKGWPLSKMDIGVPLLAIGCYSQVNPITLTAALLLLVA 116
DENV-4          ENTSANLSLAAIANQAAVLMGLGKGWPLHRMDLGVPLLAMGCYSQVNPTTLIASLVMLLV 113
JEV             TSEYVTTSLASINSQAGSLFVLPRGVPFTDLDLTVGLVFLGCWGQITLTTFLTAMVLATL 120
WNV             TSDYINTSLTSINVQASALFTLARGFPFVDVGVSALLLAAGCWGQVTLTVTVTAATLLFC 120
                 .   . **::*  **  *: * :* *:  :.: . *:  **:.*:.  .  ::  :

DENV-1          HYAIIGPGLQAKATREAQKRTAAGIMKNPTVDGIVAIDLDPVV-YDAKFEKQLGQIMLLI 176
DENV-3          HYAIIGPGLQAKATREAQKRTAAGIMKNPTVDGIMTIDLDPVI-YDSKFEKQLGQVMLLV 175
DENV-2          HYAIIGPGLQAKATREAQKRAAAGIMKNPTVDGITVIDLEPIP-YDPKFEKQLGQVMLLV 175
DENV-4          HYAIIGPGLQAKATREAQKRTAAGIMKNPTVDGITVIDLEPIS-YDPKFEKQLGQVMLLV 172
JEV             HYGYMLPGWQAEALRAAQRRTAAGIMKNAVVDGMVATDVPELERTTPLMQKKVGQVLLIG 180
WNV             HYAYMVPGWQAEAMRSAQRRTAAGIMKNAVVDGIVATDVPELERTTPIMQKKVGQIMLIL 180
                **. : ** **:* * **:*:*******..***: . *:  :    . ::*::**::*:

DENV-1          LCTSQILLMRTTWALCESITLATGPLTTLWEGSPGKFWNTTIAVSMANIFRGSYLAGAGL 236
DENV-3          LCAVQLLLMRTSWALCEVLTLATGPITTLWEGSPGKFWNTTIAVSMANIFRGSYLAGAGL 235
DENV-2          LCVTQVLMMRTTWALCEALTLATGPISTLWEGNPGKFWNTTIAVSMANIFRGSYLAGAGL 235
DENV-4          LCAGQLLLMRTTWAFCEVLTLATGPVLTLWEGNPGRFWNTTIAVSTANIFRGSYLAGAGL 232
JEV             VSVAAFLVNPNVTTVREAGVLVTAATLTLWDNGASAVWNSTTATGLCHVMRGSYLAGGSI 240
WNV             VSLAAVVVNPSVKTVREAGILITAAAVTLWENGASSVWNATTAIGLCHIMRGGWLSCLSI 240
                :.   .::  .  :. *   * *..  ***:.... .**:* * . .:::**.:*:  .:

DENV-1          AFSLMKSLGGG--RR--- 249
DENV-3          ALSIMKSVGTG--KR--- 248
DENV-2          LFSIMRNTTTA--RR--- 248
DENV-4          AFSLIKNAQTP--RRGTG 248
JEV             AWTLIKNADKPSLKR--- 255
WNV             TWTLIKNMEKPGLKR--- 255
                  ::::.      :*
```

Figure S3. Multiple sequence alignment of the primary amino acid sequence of NS4B of different flaviviruses. Complete NS4B amino acid alignments of the selected flaviviruses used in our study showing conserved regions within the DENV serotypes, WNV, and JEV as depicted by the boxed residues. Virus abbreviations (Genbank accession number: DENV-1: Dengue 1 (EU848545.1); DENV-2: Dengue 2 (EF457904.1); DENV-3: Dengue 3 (M93130.1); DENV-4: Dengue 4 (AY947539.1); WNV: West Nile virus NY99 strain (YP_001527886); JEV: Japanese encephalitis (NP_775673).
